# Using implementation science frameworks to explore barriers and facilitators for parents’ use of therapeutic strategies following a parent-mediated autism intervention

**DOI:** 10.1177/13623613221125630

**Published:** 2022-10-31

**Authors:** Sophie Carruthers, Natasha Mleczko, Stephanie Page, Shalini Ahuja, Ceri Ellis, Patricia Howlin, Kathy Leadbitter, Lauren Taylor, Vicky Slonims, Tony Charman

**Affiliations:** 1 King’s College London, UK; 2 University of Manchester, UK; 3 University of Western Australia, Australia; 4 Guy’s and St Thomas’ NHS Foundation Trust, UK

**Keywords:** autism spectrum disorders, interventions – psychosocial/behavioural, qualitative research

## Abstract

One core component of the Paediatric Autism Communication Therapy–Generalised involves supporting parents to change their interaction and communication style with their child. This behaviour change has been found to affect child outcomes. Implementation science methodologies offer a range of opportunities to investigate how interventions are delivered in practice; however, few autism intervention studies have used such designs to explore the behaviour change of parents. We interviewed 27 parents and explored their use of intervention strategies after the Paediatric Autism Communication Therapy–Generalised trial. We employed the Theoretical Domains Framework and Consolidated Framework for Implementation Research to systematically explore a range of barriers and facilitators experienced by the parents associated with parents’ characteristics, their context and features of the intervention. Our analysis revealed barriers and facilitators across three themes: Motivating Factors, which was further subdivided into Compatibility and Buy-In and Alignment of Goals and Outcomes; Opportunity and Support; Parent Characteristics. Almost all parents reported continued use of the Paediatric Autism Communication Therapy–Generalised strategies. Facilitators (e.g. parental confidence in using the strategies) and barriers (e.g. child’s behaviour) were identified. Consideration of these factors can inform identification of implementation strategies to test in future studies of Paediatric Autism Communication Therapy–Generalised and other parent-mediated early autism interventions.

**Lay abstract:**

Many early autism interventions teach parents therapeutic strategies to help them adjust their communication style with their children. Research has shown that this behaviour change in parents leads to improvements in child communication. It is, therefore, important to learn what factors support or hinder parents in their use of therapeutic strategies learned in such interventions. This study set out to interview parents who had participated in a research trial of the Paediatric Autism Communication Therapy–Generalised intervention. We interviewed 27 caregivers and explored their use of the strategies up to 2 years after the end of the research trial. Qualitative frameworks were used to inform interview questions and data analysis. These frameworks focused on a range of contextual factors, including parents’ characteristics, their context and features of the intervention. Parents reported barriers and facilitators to using Paediatric Autism Communication Therapy–Generalised strategies across three themes: Motivating Factors; Opportunity and Support; Parent Characteristics. One of these themes, Motivating Factors, was further divided into the subthemes Compatibility and Buy-In and Alignment of Goals and Outcomes. Almost all parents reported continued use of the Paediatric Autism Communication Therapy–Generalised strategies. Facilitators included parental confidence in using the strategies and barriers included child’s behaviour. Consideration of these factors can inform ways to better support parents in future autism interventions.

## Introduction

Use of evidence-based autism interventions in community services is limited (Brookman-Frazee et al., 2010; Dingfelder & Mandell, 2011) and, where they are used, effect sizes tend to be substantially smaller in the community compared to the outcomes achieved in efficacy trials (Nahmias et al., 2019). One factor that may contribute to this research–practice gap is the use of methodologies within trials that rarely draw on the experience of practitioners, autistic individuals and their families (Guldberg, 2016). This contribution may be particularly important in relation to parent-mediated interventions, a therapeutic approach with growing evidence for supporting young autistic children (Nevill et al., 2018; Oono et al., 2013). Such approaches are attractive as parents are anticipated to use the techniques over long-time periods and across many different situations, thereby providing a large ‘dose’ of intervention and support for generalisation and maintenance of children’s skills.

In the autism field, these approaches often involve parents being supported to use interaction styles, such as being synchronous (i.e. following the child’s lead), that have been evidenced to improve children’s communication (Siller & Sigman, 2008). Examples include the parent-mediated versions of Naturalistic Developmental Behavioural Interventions (NDBI) such as Joint Attention, Symbolic Play and Emotional Regulation (JASPER) therapy (Kasari et al., 2015), and developmental approaches such as Preschool Autism Communication Therapy (PACT) (Green et al., 2010) and Hanen More Than Words (Carter et al., 2011). Reviews and meta-analyses of parent-mediated interventions show evidence of small improvements in children’s communication (Nevill et al., 2018; Oono et al., 2013), core autism characteristics (Nevill et al., 2018; Oono et al., 2013), socialisation and cognition (Nevill et al., 2018) and larger effects for parent interaction behaviours (Oono et al., 2013). As parents develop the skills and understanding of how to adjust their communication for their child, such approaches have potential benefits for both the child and parent (Estes et al., 2019).

These interventions require parents to change the ways in which they interact with their child (Rogers et al., 2020; Stahmer et al., 2017). Mediation studies of two intervention trials indicated that the change in parental behaviour was the key driver of improvement in child outcomes (Gulsrud et al., 2016; Pickles et al., 2015; Shih et al., 2021). Furthermore, larger gains in child outcomes are positively associated with parents’ mastery of the intervention strategies (Pickles et al., 2015; Shire et al., 2015). Therefore, the level of parents’ learning and fidelity is key. In the PACT trial, although the mean proportion of parental interaction rated as synchronous among those who received the intervention increased from 32% at baseline to 52% at endpoint, there was considerable variation in the level attained (Pickles et al., 2015). One interpretation is that some parents could be better supported to increase their mastery and use of the strategies, which may have knock-on benefits for their children’s outcomes.

An important step in supporting behaviour change is to identify barriers and facilitators that individuals experience in their efforts to implement that behaviour. Existing research has begun to highlight several factors that can influence parents’ ability to use parent-mediated autism interventions. First, parents have highlighted certain aspects of the intervention itself that can facilitate their use of strategies, such as techniques that are easily integrated into daily routines (McConnell et al., 2015; Pickard et al., 2016), strong parent-therapist alliance (Johnson & Hastings, 2002; Pickard et al., 2016), flexible training approaches (Pickard et al., 2016; Raulston et al., 2018) and feedback on progress (Raulston et al., 2018). Parents also highlight personal factors, such as their level of confidence in the therapy (Moore & Symons, 2011; Solish & Perry, 2008), belief in their own capabilities (i.e. self-efficacy; Moore & Symons, 2011; Solish & Perry, 2008), their knowledge of autism (Solish & Perry, 2008), their available time (Johnson & Hastings, 2002; Leadbitter et al., 2020) and the extent of family and socioemotional support (Johnson & Hastings, 2002; Pickard et al., 2016; Raulston et al., 2018). Seeing and understanding progress in their child can also facilitate implementation (Carr & Lord, 2016; Johnson & Hastings, 2002; Leadbitter et al., 2020). Finally, some parents identified challenges in implementing the strategies at home if it was not something their child was interested in doing (Carr & Lord, 2016) or when their child was tired or not concentrating (Johnson & Hastings, 2002).

While these findings highlight several barriers and facilitators, most of these studies have relied on open questions about the parents’ general experience of the intervention, rather than systematically exploring the factors influencing implementation. Without a theoretical framework, there is a risk of overlooking key aspects of the implementation context (Atkins et al., 2017; Powell et al., 2017). A framework also offers links to underlying theories of behaviour change (Michie et al., 2011). Across healthcare research, commonly used frameworks to explore barriers and facilitators include the Theoretical Domains Framework (TDF; Cane et al., 2012) and the Consolidated Framework for Implementation Research (CFIR; Damschroder et al., 2009). These frameworks were initially developed for considering the behaviour of health professionals related to implementation of evidence-based practices, and they consider a broad range of contextual factors that can influence behaviour. The use of implementation science frameworks within the autism field is increasing (Broder-Fingert et al., 2019; Brookman-Frazee, Chlebowski, Suhrheinrich, et al., 2020), but there are very few examples of using these frameworks with parents (Rieth et al., 2018). The TDF (TDF v2; Cane et al., 2012) was selected to design the topic guide for this study because of its focus on personal characteristics (e.g. skills, goals and intentions) which have not been widely studied in the context of parent-mediated autism interventions (Siller et al., 2018; Trembath et al., 2019). The CFIR was used as a second framework alongside the TDF to strengthen the coding in relation to the wider context, for example, characteristics of the intervention.

In order to maximally support children’s generalisation and maintenance of their communication skills, continued use of the strategies after formal therapy ends is assumed to be advantageous (i.e. sustainability of the behaviour change; Moore et al., 2017). In a follow-up study of PACT, improvements in child initiations were maintained, but parents, on average, were less synchronous during interactions with their children than at trial endpoint 6 years previously (Pickles et al., 2016). Gaining insight into the extent of sustained use of the therapy, or how it evolves over time, will provide better understanding of the mechanisms underpinning longer term outcomes.

This study focuses on the Paediatric Autism Communication Trial–Generalised (PACT-G; Green et al., 2018), a parent-mediated therapy based on developmental principles, which supports parents and teaching assistants to become more synchronous with their child. In this study, we aimed to investigate parents’ perspectives on factors that represented barriers and facilitators to their use of PACT-G strategies in the immediate years after the completion of therapy. Specifically, this study aimed to identify facilitators and barriers to the ongoing implementation of the PACT-G therapeutic strategies.

## Methods

The Consolidated Criteria for Reporting Qualitative Studies (COREQ; Tong et al., 2007) were used to analyse study findings.

### Participants

A total of 27 caregivers (26 parents, 1 grandparent) participated, each of whom had finished the PACT-G trial between 14 and 28 months earlier. Participants were drawn from the 41 caregivers who participated in the intervention arm of the trial in London. Parents received a letter informing them of the study, which was followed up with a phone call or an email inviting them to participate. Of 37 caregivers initially invited to participate, 28 agreed; 1 did not complete the interview due to illness, 6 declined and 3 were uncontactable. In order to ensure a representative subsample of the PACT-G families and therapy experience, potential participants were grouped by child’s age group (preschool vs school age at trial baseline), socioeconomic status, and level of engagement during the therapy (see Table 1), and then randomly ordered such that invited participants were balanced across these variables. Due to practical constraints, data coding could not commence prior to the end of data collection. This prevented the use of theme saturation to inform stopping point. The team, therefore, used guidance from the literature on which factors to consider when making this decision. Further detail is outlined in Supplementary Materials. Participants each received a £20 voucher. Table 1 presents a summary of participant characteristics.

**Table 1.** Summary of sample characteristics.

	*N* (%)	Mean	SD	Minimum	Maximum
Child age (years)		5.10	2.11	2.23	11.0
Parent age (years)		37.7	6.26	32.2	60.2
Parent relationship with child					
Biological mother	23 (85.2%)				
Biological father	3 (11.1%)				
Biological grandparent	1 (3.7%)				
Child ethnicity					
White	10 (37.0%)				
Asian	2 (7.4%)				
Black	8 (29.6%)				
More than one race	5 (18.5%)				
Other	2 (7.4%)				
Parent ethnicity					
White	9 (31.0%)				
Asian	2 (7.4%)				
Black	8 (29.6%)				
More than one race	5 (18.5%)				
Other	2 (7.4%)				
Not reported	1 (3.7%)				
Adults in home		1.93	0.68	1	4
Children in home		1.93	0.78	1	4
Marital status					
Single	4 (14.8%)				
Married/civil partnership	17 (63%)				
Co-habiting	5 (18.5%)				
Divorced	1 (3.7%)				
Parent education^a^	25 (90.5%)				
Parent socioeconomic status^b^	16 (59.3%)				
Number of therapy sessions^c^		10.6	1.69	7	12
Engagement during therapy sessions (range = 1–3)^d^		2.54	0.34	1	3
Months since endpoint assessment		19.7	3.31	14.9	28.0

SD: standard deviation.

*N* = 27.

Numbers of adults and children living in the home were updated at the time of the interviews; all other data were collected at the time of the PACT-G trial.

a Defined as ⩾1 parent educated to post-16 education.

b Defined as ⩾1 parent in a professional or administrative occupation versus all others.

c *N* acceptable sessions as rated by therapists.

d Rated by therapists during each attended session except the first: 1 (not engaged) to 3 (fully engaged).

### PACT-G

The PACT-G intervention aims to increase parental and teaching assistant synchrony with their children through a range of strategies (for details, see Supplementary Table S1). Families with children aged 2–11 years with a diagnosis of autism were eligible to participate in the trial. Parents were required to communicate with their child at least partially in English in the home and be willing and able to film parent–child interaction in English. They received up to 12 intervention sessions delivered in the home and over video conference and were also invited to attend up to six home-school meetings to discuss the therapy with the teaching assistant (exploration of the implementation within the school is presented elsewhere; Ellis, 2020). Parents were asked to make time to practice therapy strategies daily for half an hour. The intervention was a free-to-access provision via the UK National Health Service (for further details of trial inclusion/exclusion criteria, see Green et al., 2018). The PACT-G trial and this study received ethical approval from the North West – Greater Manchester Central Research Ethics Committee. This study was completed prior to publication of the trial results.

### Procedure

We used semi-structured telephone interviews. The topic guide (Supplementary Table S2) was initially piloted with two parents after which some small adjustments were made to the phrasing of questions. As the adjustments were minor, data from the pilots were included in the final analysis. Interviews were conducted by three female MSc or PhD researchers (N.M., S.P., S.C.) with training in qualitative interviewing. Parents were informed that the interviewers were interested in exploring their experience of using the strategies since the trial and factors that influenced their use. As data collection occurred during the first national COVID-19 lockdown, parents were encouraged to reflect on their experience before the lockdown but were informed that they were welcome to reflect on how things were different in the current circumstances. Interviews were audio-recorded and took on average 46 min (range = 19–74 min).

### Frameworks

The interview questions reflected all 14 subdomains of the TDF (TDF v2; Cane et al., 2012) (see Supplementary Table S2). The TDF was devised as an integrative framework of theories of behaviour change, and each subdomain is considered to represent a key determinant of behaviour (i.e. either a barrier or a facilitator; Cane et al., 2012).

**Figure 1.**
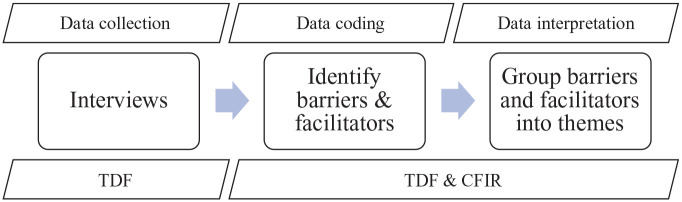
A schematic outlining the use of frameworks and models during the study. CFIR: Consolidated Framework for Implementation Research; TDF: Theoretical Domains Framework.

Ahead of coding, we introduced the CFIR as a second framework to be used alongside the TDF because the contextual analysis, which was key to this work, was inadequately captured by the ‘Environmental Context and Resources’ domain of the TDF. We selected the Intervention Characteristics and Outer Setting domains from the CFIR (see Supplementary Table S4; Damschroder et al., 2009). The Intervention Characteristics domain comprises eight subdomains that explore how elements of the therapy might influence ease of implementation, such as how adaptable and complex it is. As the CFIR was designed for use with health professionals (Kirk et al., 2015), the Outer Setting domain covers the patient and their needs, external policy influences and organisational networks. For this study, the Outer Setting subdomains were replaced with codes for Child’s School and Child’s Needs, in line with similar adaptations reported elsewhere (Norman et al., 2019). The TDF and CFIR are syntheses of behaviour change theories and are often used together effectively to explore implementation (Birken et al., 2017). The use of frameworks and models across the study design is outlined in Figure 1. Definitions for key implementation terminology are provided in Supplementary Materials.

### Data analysis

We followed guidance for TDF studies as outlined in Atkins et al. (2017). Interviews were transcribed verbatim and transcripts uploaded to NVivo 12. S.C., N.M. and S.P. familiarised themselves with the data. They coded three transcripts together to develop the coding framework (Supplementary Tables S3 and S4) before dividing the remaining coding between them. Coders were consistent with each other in their coding before independent coding started. In addition to the TDF and CFIR codes, an additional code was used to capture details of strategy use. Data were coded inductively and deductively (see Supplementary Table S4 for deductive codes). Guided by Braun and Clarke (2013), quantitative reliability measures were not used as they were judged not to be appropriate criteria for this study. We were inclusive in our coding and, where relevant, could choose to code an utterance under more than one code. If there were disagreements between coders, S.C. made a final decision. Across coding and subsequent stages, S.C., N.M. and S.P. collaborated closely, with regular discussion to check that utterances were captured under all applicable codes. S.C. reviewed coded transcripts to ensure consistency.

After coding, we generated the statements that represented either barriers or facilitators. The most salient barriers and facilitators were identified and grouped into overarching themes. As per previous TDF studies, salient barriers and facilitators were identified by the following criteria: high frequency of parent report, a presence of conflicting themes within/across domains and/or evidence that they influenced strategy use. Those with lower frequency counts but potential clinical importance were also considered (Atkins et al., 2017). To support transparency during the process of analysis, we used Iterative Categorisation, a set of standardised steps that results in an audit trail to evidence each stage of the process (Neale, 2016). Community members were not involved in the analysis of this study.

### Identifying implementation strategies

As a final stage of this study, we used an online tool to identify potential implementation strategies that could be helpful in addressing the barriers identified by parents. The CFIR–Expert Recommendations for Implementing Change compilation (ERIC) mapping tool (Waltz et al., 2019) matches barriers to implementation strategies that could overcome them. The tool is publicly available at www.cfirguide.org. It was developed by asking implementation science experts to rank the top seven implementation strategies that would address each CFIR barrier. For further details, see Waltz et al. (2019). Further details of how we used the tool in this study are provided in Supplementary Materials.

## Results

### Use of strategies

Most parents stated that they still used PACT-G strategies, with a majority adhering to the central tenet, that is, to ‘follow the child’s lead’. Many parents reported using the strategies regularly during a typical week. Around a third of parents told us they did not use the strategies as much as they would like. A few parents reported that they no longer used the strategies. Reasons for stopping included parent fatigue, mismatch of goals between parent and therapy, and child improvement in social communication skills.

### Key themes

**Figure 2.**
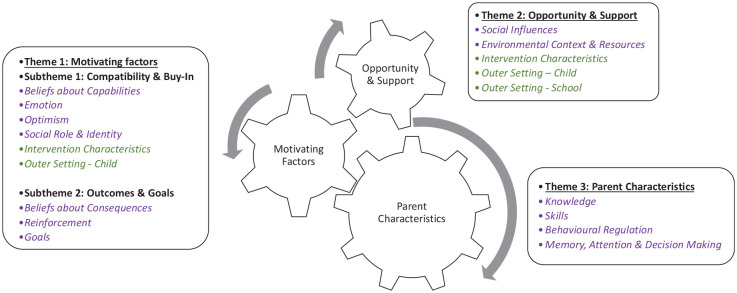
Summary of themes including theoretical Domains Framework (purple) and Consolidated Framework for Implementation Research (green) domains associated with the barriers and facilitators within each theme.

Barriers and facilitators were grouped within three overarching themes: Motivating Factors; Opportunity and Support; Parent Characteristics (Figure 2 and Tables 2 and 3). Quotes for each barrier and facilitators are provided in Supplementary Tables S5–S7.

**Table 2.** A summary of the facilitators grouped by theme and TDF/CFIR subdomain with parent report frequency.

TDF or CFIR subdomain	Facilitators	Frequency
Motivating Factors: Compatibility and Buy-In		
Beliefs about capabilities	I’m confident using the strategies	23
Emotion	I feel very positive about the PACT-G strategies	22
Relative advantage	PACT-G strategies have advantages over other therapies	17
Social role and identity	Training parents to use the strategies with their children is the right approach	17
Optimism	I believe in the principles of PACT-G	14
Design quality and packaging	Video feedback provided me with a secure understanding and knowledge of the strategies^a^	16
	The therapists were wonderful teachers and provided a lot of support^a^	10
Motivating Factors: Alignment of Goals and Outcomes		
Goals	I want my child to communicate better	17
	I want my child to be more independent and have a better quality of life	10
	Using the PACT-G strategies is still a top priority for me	5
	I wanted to learn how to support my child (independently from professionals)^a^	5
Beliefs about consequences	My relationship with my child has improved	25
	I have learned about my child and their autism	23
	My child is more confident and engaged with things around him or her	21
	My wellbeing has improved	21
	My child’s wellbeing has improved	16
	My child’s communication has improved	16
	The wider family benefits from me using the strategies	7
Reinforcement	Seeing the progress in my child makes me want to continue using the strategies	23
Opportunity and Support		
Complexity	The strategies were easy to learn^a^	21
School	I enjoyed working with the school^a^	17
Child	I can incorporate my child’s interests when using the strategies	12
Social influences	My family and friends are supportive of using PACT-G strategies	12
Adaptability	I can easily adapt the strategies to suit our needs or the situation	10
Parental characteristics		
Knowledge	I understand the philosophy of PACT-G	19
Skills	Being patient is important	17
	The strategies are now automatic and spontaneous	13
	I’m open to thinking about how to change my behaviour	10
	Self-motivation is important	8
Behavioural regulation	I plan when I’m going to use the strategies	7

CFIR: Consolidated Framework for Implementation Research; TDF: Theoretical Domains Framework; PACT-G: Paediatric Autism Communication Trial–Generalised.

a These facilitators may be more closely associated with implementation at the time of the therapy but may have consequences for later use of strategies.

**Table 3.** A summary of the barriers grouped by theme and TDF/CFIR subdomain with parent report frequency.

TDF or CFIR subdomain	Barriers	Frequency
Motivating Factors: Compatibility and Buy-In		
Optimism	You need to persevere because progress can be slow	8
Emotion	I wasn’t so positive about the therapy at the start^a^	8
Social role and identity	Some strategies go against my natural parenting instincts	5
Beliefs about capabilities	I lack confidence using the strategies	4
Child	PACT-G is too easy for my child	2
Relative advantage	Other therapies are more suitable for my child	2
Motivating Factors: Alignment of Goals and Outcomes		
Goals	PACT-G is less of a priority for me now	9
Child	My child has other needs that demand my energy and attention	8
Opportunity and support		
Child	My child’s behaviour and mood influences when I can use the strategies	20
	I need to accommodate my child’s specific needs	13
Environmental context and resources	I need time to do the strategies, which I don’t always have spare	17
Social influences	My family/friends do not get involved in using the strategies	13
School	I didn’t have a strong relationship with the school at the time of the trial^a^	8
Parental characteristics		
Memory, attention and decision processes	Sometimes I forget to use the strategies	7
Behaviour regulation	The strategies sometimes require conscious effort and thought	7

CFIR: Consolidated Framework for Implementation Research; TDF: Theoretical Domains Framework.

a These barriers may be more closely associated with implementation at the time of the therapy but may have consequences for later use of strategies.

#### Motivating factors

Within the overarching theme of Motivating Factors, there are two subthemes: ‘Compatibility and Buy-In’ and ‘Alignment of Goals and Outcomes’.

##### Compatibility and buy-in

This subtheme represents the compatibility and commitment parents feel towards the PACT-G therapeutic style and philosophy. If parents are compatible with the approach, this was reflected in their attitudes and beliefs towards the therapy.

The majority of parents reported feeling confident in using the strategies (*TDF Beliefs about Capabilities*) and very positive about them (*TDF Emotion*). Some parents reflected on how PACT-G had come along at a difficult time in their lives (e.g. shortly after the child’s diagnosis) and provided them with support and empowerment. This substantially positive emotion was often accompanied by belief in the philosophy and principles of the therapy (*TDF Optimism*):

I strongly believe in [PACT-G] because follow the lead is so important, it’s crucial in life because you give them . . . confidence. (P19)

Several aspects of the therapy itself were raised during the interviews. Many parents reported that the PACT-G strategies had advantages over other therapeutic approaches (*CFIR Intervention Characteristics – Relative Advantage*). The video feedback component of the training was mentioned by many as a powerful learning technique; this provided a strong foundation for parents’ learning which facilitated sustained strategy implementation (*CFIR Intervention Characteristics – Design Quality & Packaging*). Therapists themselves were also commended for being highly supportive (*CFIR Intervention Characteristics – Design Quality & Packaging*). Furthermore, many parents believed that being trained to deliver the strategies themselves with their children was a specific strength of the approach (*TDF Social Role & Identity*).

Six barriers were also identified within this subtheme. First, some parents reflected on how the therapy was ‘difficult in the beginning’ (*TDF Emotion*). Although they eventually came to understand the value of it, some initially found the parent-mediation difficult, as they felt uncertainty about taking on the role of a therapist. Others suggested that having to change their interaction style with their child was a challenge emotionally, as well as practically, as they worried that they had previously been communicating with their child in the wrong way:

I struggled [to take on the role of the therapist] in the beginning. Where I come from a child has . . . one-to-one sessions with a speech and language therapist. So, in the beginning I was not happy with it. But . . . at the end I think it is the right way because . . . the first therapist living with the child is the parent . . . If the therapist is doing one hour a week with good communication and then the rest of the week nothing is happening . . . it does not create consistency, it just confuses the child. (P4)

Some parents reported additional obstacles and challenges. For example, some parents reported that their child’s progress was slow, particularly at first, requiring parents to remain positive and persevere (*TDF Optimism*). In addition, a few parents felt that the strategies went against their natural parenting instincts, such as wanting to intervene and teach their child (*TDF Social Role & Identity*):

I always try not to correct him too quickly, because we focused on that a lot during the therapy . . . let him take the lead. If he does something incorrectly . . . your instinct is to correct and teach. (P22)

Furthermore, a handful of parents reported lacking confidence in how to use the strategies (*TDF Beliefs about Capabilities*). Two further barriers, reported by a minority of parents, represent important factors that were raised by those who had either stopped using the strategies or were considering seeking other therapies. For a small number of parents, PACT-G did not meet the needs of the child as they felt their child could, and should, be stretched further (*CFIR Outer Setting – Child*). These parents thought that Applied Behaviour Analysis and Pivotal Response Training were likely to be more effective (*CFIR Intervention Characteristics – Relative Advantage*).

##### Alignment of goals and outcomes

Where their goals were realised in the outcomes they were observing, parents reported that it was this progress that sustained their implementation of the strategies (*TDF Reinforcement*). In this way, this subtheme represents the factors that resulted in ongoing and sustained motivation to implement the therapy.

Regarding their children, many parents reported them to be more confident and engaged in daily life (*TDF Beliefs about Consequences*). For instance, this included children engaging with and enjoying play with their parents more and being more confident around other people. In line with the goals of the therapy, many parents reported improvements in their child’s communication and in their child’s wellbeing (*TDF Beliefs about Consequences*):

To be more in tune with him helps him being less stressed, so he felt a bit more understood . . . I think there’s less frustration on his part . . . when he’s home, he has . . . less tantrums, he’s not quite as aggressive. (P1)

Parents also described a range of benefits related to themselves (*TDF Beliefs about Consequences*). Many felt the strategies had improved their relationship with their child, and helped them to learn a lot about their child and their child’s autism. For instance, some felt that through the strategies, they had learned to let go of and accept things about their child’s autism diagnosis:

I remember he had a . . . pretend cake. When we started [PACT-G] I was desperately trying to get him to slice this cake . . . trying to make him be the child that I wanted him to be. All he wanted to do was tap the underside of the cake . . . so the therapist said . . . ‘tap it with him’. When I was trying to get him to slice the cake, he was just . . . pushing me away . . . but when I tapped the underside of the cake with him . . . he was totally engaged with me and he was looking at me, laughing, and he enjoyed it . . . I realised . . . he doesn’t have to do things the way I think he should do them . . . it doesn’t mean that it’s not playing properly. (P25)

Many of the same parents said that their own wellbeing had improved, which they linked to their improved relationship with their child and because they spent less time worrying about their child’s autism and communication (*TDF Beliefs about Consequences*). Finally, several parents also identified benefits for the wider family (*TDF Beliefs about Consequences*):

I think one of the blockers for me was that I was just too anxious about trying to get my child to talk and I think what he needed was time to just . . . do it on his own terms. I think PACT-G helped me win over that anxiety. There’s [now] a lot less anxiety on my part and I think my child can feel it as well because [our relationship] becomes a lot more relaxed. (P6)

The facilitating force of these outcomes is likely, in part, due to their close alignment with parents’ goals. When asked about what they wanted from the therapy, the most common response was to improve their child’s communication (*TDF Goals*). More broadly, parents also reported wanting to learn how to support their child independently of professionals and wanting their child to have a better quality of life and be more independent (*TDF Goals*). A number of parents identified that using PACT-G strategies remained a top priority for them (*TDF Goals*).

Two barriers were identified within this theme. First, some parents reported that using PACT-G strategies was no longer a top priority for them (*TDF Goals*). Sometimes this reflected their children making sufficient progress so that parents felt less urgency to use the strategies. Other parents reported competing priorities. These parents reflected on using the strategies less often, particularly during times when their child had needs that were a higher priority than communication, including difficulties with behaviour, sleep, toileting and eating (*CFIR Outer Setting – Child*).

#### Opportunity and support

This theme focuses on factors that influence the opportunities parents have to use the strategies and how easy that is to do, including the presence of support from others, the availability of time and the child’s needs. How easy new behaviours are to learn and use is important for behaviour change. The majority of parents noted that the strategies were easy to learn (*CFIR Intervention Characteristics – Complexity*). Around half of parents told us how incorporating their child’s interests into the strategies made implementation easier (*CFIR Outer Setting – Child*). A number of parents also reported that the strategies were adaptable, meaning that they could be adjusted to meet the changing needs of their child and the flexibility of the strategies made it possible to use them across different situations (*CFIR Intervention Characteristics – Adaptability*):

It was something that you can take away and apply to most situations, and it was simplistic enough to incorporate into your daily lives. (P10)

Support from others is also a key factor in behaviour change. The majority of parents enjoyed working alongside the teaching assistant in learning and using the strategies (*CFIR Outer Setting – School*). Some parents also reported receiving support from family and friends regarding use of the strategies (*TDF Social Influences*).

This theme was also associated with a number of barriers. A majority of parents reported difficulties in finding the time to use the strategies, because of other demands on their time such as caring for other children (*TDF Environmental Context & Resources*):

It’s just you have to be determined to learn it as well because sometimes you can be quite busy with other children and so [you need to] take that time out to do it. Sometimes it isn’t easy. (P26)

With regard to support, around a third of parents did not have a strong relationship with the school when learning to use the strategies (*CFIR Outer Setting – School*). Around half of the parents reported that family and friends were not involved or did not know about the strategies (*TDF Social Influences*).

Finally, a number of barriers related to the needs of their child (*CFIR Outer Setting – Child*). Three quarters of the parents told us that their child’s behaviour and mood influenced if, when and how they could use the strategies. Moreover, around half of parents reported needing to accommodate their child’s specific preferences when considering how and when to use the strategies, such as whether or not other people were present.

#### Parent characteristics

The majority of parents evidenced a clear understanding and knowledge of the PACT-G principles and philosophy (*TDF Knowledge*). Parents reflected on how this knowledge had secondary consequences. For instance, some reported that learning about PACT-G shifted their goals, for example, from wanting their child to be verbally fluent to aiming for any form of communication even if nonverbal. Other parents told us that being trained in PACT-G helped them learn about autism which, in turn, supported them to accept and worry less about it. Other consequences included understanding their child better and reconsidering what they could do as a parent to support their child’s development.

With practice, this knowledge translated into the strategies becoming automatic and spontaneous for around half of the parents interviewed (*TDF Skills*). Others told us that they plan when to use the strategies, including during particular activities or times of the day (*TDF Behavioural Regulation*). Parents also reflected on a number of attributes and skills that they felt facilitated their use of the strategies, including having patience, being open to learning how to change their behaviour and being self-motivated (*TDF Skills*).

In terms of barriers, several parents reported forgetting to use the strategies, which some linked to the absence of scheduled reminders or therapy sessions after the trial had ended (*TDF Memory, Attention & Decision Processes*):

It’s really easy but when you want to do it and you have an autistic child, it’s a challenge. You constantly forget and you need someone to remind you to do that. (P25)

Finally, a number of parents reported that conscious effort and thought were required to use the strategies, for instance, they needed to remind themselves to step back and not be directive (*TDF Behavioural Regulation*).

### Implementation strategies

The 10 highest ranked implementation strategies suggested by the CFIR–ERIC mapping tool are outlined in Supplementary Table S8. After considering priority barriers and the context of parent-mediated therapies, we identified the following implementation strategies as target areas for further exploration:

- Assess for readiness and identify barriers and facilitators;
- Conduct local needs assessment;
- Promote network weaving and
- Conduct educational meetings.

Supplementary Table S9 outlines definitions for these four strategies alongside examples of the barriers that they may help to overcome.

## Discussion

This study applied implementation science frameworks to characterise barriers and facilitators of parents’ implementation of PACT-G strategies up to 28 months after the trial endpoint. Almost all parents interviewed continued to use the strategies in some way, particularly with regard to ‘follow the child’s lead’, the overarching principle that encompasses the range of strategies that therapists tailor for each family. We found that around a third would have liked to use them more often, and a few had stopped altogether. Therefore, the majority of parents continued to want to implement the strategies, but, at least for some, barriers were experienced in achieving the desired level of implementation.

We identified three overarching themes of barriers and facilitators. The first subtheme of Motivating Factors, Compatibility and Buy-In, showed that most parents continued to feel very positive about the strategies. They remarked on aspects of the PACT-G delivery that supported their initial learning, which increased confidence in using the strategies and supported their ongoing use. Certain facilitators such as a supportive therapist, having confidence in the therapy, and belief in their own capabilities echoed parents’ views in previous studies (Johnson & Hastings, 2002; Moore & Symons, 2011; Pickard et al., 2016; Solish & Perry, 2008). An important component of motivation in the context of parent-mediated interventions is the extent to which parents are willing to take on the ‘therapist’ role for their child. In this study, the majority of parents agreed with the benefits of this approach, although some parents took time to see its full value. There are several reasons why parents may be initially uncertain about a parent-mediated approach. For instance, they may perceive parent-mediated approaches to result from service cutbacks or policies that deny their child intervention delivered by expert therapists. Alternatively, parents may see the potential value, but not feel equipped with the requisite skills and knowledge and be unsure as to whether they will acquire them. Different therapeutic approaches will suit parents to varying degrees, including over time. As children get older, their needs may shift, potentially requiring different parenting styles (Zarra-Nezhad et al., 2014). A couple of parents reported seeking alternative therapies instead of PACT-G. This compatibility element is an important consideration for implementation. Currently, there is limited research on compatibility between parent and therapeutic approach within the context of autism (Karst & Van Hecke, 2012; Wainer et al., 2017).

The second subtheme of Motivating Factors was Alignment of Goals and Outcomes. Parents reported various positive changes in their children that they attributed, at least in part, to PACT-G. These benefits were salient facilitators, similar to those reported elsewhere (Carr & Lord, 2016; Johnson & Hastings, 2002; Leadbitter et al., 2020). Such improvements act as reinforcement and sustain motivation to continue using the therapy (Stahmer et al., 2017). As a therapy aimed at improving children’s communication, it is noteworthy that an equally high proportion of parents noted improvement in their child’s wellbeing and behaviour as in their communication, with parents suggesting that lower levels of frustration for their children may be a downstream effect of the change in parent’s behaviour and their improved understanding of their child. This is consistent with recent evidence indicating that improvements in parents’ sense of competence mediate the relationship between parent training and child outcomes (Brookman-Frazee, Chlebowski, Villodas, et al., 2020). Parents also reported benefits for their own wellbeing, in line with previous findings (Da Paz & Wallander, 2017; Estes et al., 2019).

Within the theme of *Opportunity and Support*, the most frequently reported barriers were associated with child behaviour and mood, which limited opportunities to use the strategies. Being able to tailor the strategies (e.g. including the child’s interests) and adapt them across situations are aspects of the PACT-G approach aimed at addressing these kinds of barriers; however, such issues remained a challenge for many parents. The importance of having opportunities and support to use the strategies has also been reported by parents in previous studies (Johnson & Hastings, 2002; Leadbitter et al., 2020; McConnell et al., 2015; Pickard et al., 2016; Raulston et al., 2018).

The final theme was *Parent Characteristics*. While half the parents in this study reported that the strategies had become automatic and spontaneous, some planned how and when to use them, and some reported forgetting to use them and needing to consciously think about it. From this study, it is not possible to understand what may underpin these differences, but it is possible that differing parenting styles at baseline or variable opportunities to practice may play a role. Alternatively, different parents may benefit from varying styles of coaching techniques (Friedman et al., 2012). Parent-mediated interventions can be complex and can require parents to take on extensive training and expertise in order to achieve fidelity (Rogers et al., 2012). Relatively few studies have explored how parents’ capabilities, characteristics and contexts may interact with their use of therapeutic strategies, but preliminary studies have indicated the potential importance of self-efficacy, understanding of child development (Siller et al., 2014), capacity for reflection and self-evaluation (Siller et al., 2018) and parental stress (Stadnick et al., 2015). As the field seeks to understand moderators of intervention effectiveness, it will be crucial to consider how parental characteristics may influence the learning and use of techniques (Trembath et al., 2019).

### Clinical and research implications

Across the themes, several facilitators highlighted existing aspects of the PACT-G therapy that act as implementation support for parents, such as the effectiveness of a supportive interpersonal relationship with the therapist, directly involving parents in therapy, tailoring strategies for use across different situations and child interests, making the strategies easy to learn and adapt, and encouraging parents and teaching assistants to share learning. As such these represent key components of the intervention to retain as it is disseminated into community practice. While this study has focused on the PACT-G therapy, it has similarities with other parent-mediated interventions, and the views of parents interviewed in this study overlap with those of parents questioned about other approaches (Carr & Lord, 2016; Pickard et al., 2016). This study may, therefore, be informative for consideration of implementation strategies across therapeutic models.

Barriers reported in this study indicate potential opportunities to provide additional implementation support. Supported by the CFIR–ERIC tool, we identified four implementation strategies that may inform future parent-mediated interventions. For instance, parents may benefit from being introduced to other parents who have received PACT-G (i.e. *promote network weaving*). Working out how to juggle the therapy alongside other demands on their time or how to adapt the strategies to accommodate a child’s changeable behaviour may be common challenges for parents receiving the intervention. Sharing knowledge and offering encouragement to each other could overcome such barriers. It would be necessary to investigate the effectiveness of these strategies before introducing them into community practice, although some previous work lends support (Stahmer & Gist, 2001). There may also be opportunities for ‘*conducting educational meetings*’ in order to encourage greater buy-in and support from family and friends. It may also be worth exploring the value of pre-recorded talks to be watched online.

‘*Local needs assessment*’ and an ‘*assessment for readiness and identification of barriers and facilitators*’ were the final two strategies identified for future consideration. Although some assessment of families is conducted prior to referral for therapy, we posit that this could be improved for parent-mediated interventions with further research into family factors that moderate effectiveness, implementation and compatibility. In the future, it would be interesting to explore the ‘readiness’ of parents and/or the timeliness of the intervention (Jones et al., 2017; Proctor et al., 2017). For instance, it is important for clinicians to work with families to consider whether the time is right and whether a parent-mediated intervention is what they want. Further research would be needed to inform such assessment.

Currently, parents are not provided with explicit guidance around use of the strategies beyond the therapy period. This ambiguity over expectations of fidelity after the trial is important to consider in relation to our findings. The PACT-G therapy consists of six stages, the final ones being for children who have language and are starting to engage in conversations. For autistic children who do not develop fluent, reciprocal conversation, PACT-G strategies may be suitable across childhood; however, children, who progress beyond these final stages, may require more complex input. This study suggests that many parents continue to want to use the strategies in the immediate years following the therapy, but addressing a child’s changing development and communication would require some further input from a trained therapist.

### Limitations

This study has a few limitations. Families participating in research studies may be different to those who receive services in the community and the families’ experience of PACT-G within a research trial will have been different to that in the community (Rothwell, 2006; Westen et al., 2006). This study is restricted to the PACT-G therapy which is both a benefit in terms of the specificity and richness, and also a limitation in terms of the scope. By drawing a representative subsample from the diverse set of families who received the PACT-G therapy, we captured a wide range of experiences. However, as the sample were drawn from a randomised controlled trial (RCT), they had been filtered through the trial’s eligibility criteria, including willingness from an education setting to participate, which may have impacted the representativeness of the sample.

The study did not include quantitative measures of parent fidelity and implementation. We could, therefore, not link our findings to implementation outcomes (Proctor et al., 2011). While we explored a broad range of factors, we did not explore the extent to which factors such as socioeconomic status, culture or child’s age influenced the barriers and facilitators experienced. Moreover, as therapists would not have introduced all strategies to each family, nor named each strategy, we were not able to ask parents about use of specific strategies, which limited the precision with which we could report ongoing use. Member checking or community vetting was not used to confirm the interpretation, but future studies should aim to include this additional step. Parents finished the PACT-G trial between 14 and 28 months prior to the study. It is possible that over the course of this period, their memory of the experience and knowledge of the strategies diminished, thus impacting responses given in interviews. Finally, although data were collected during a COVID-19 lockdown, no systematic questioning was included to ask about the potential impact of those circumstances.

While the CFIR–ERIC mapping tool offered us a way to link the barriers reported by parents to implementation strategies, to date it has mostly been used in healthcare settings, and therefore, not all strategies were relevant to the context of this study. Mapping our 17 barriers (many of which were TDF domains) to CFIR constructs also resulted in some loss of specificity with regard to the nature of the barriers. Formal selection of strategies for use in future iterations of PACT-G should be achieved using more comprehensive methodologies (Powell et al., 2017).

## Conclusion

It has been suggested that one factor underlying the research–practice gap within the autism field is a lack of research on the experiences of families participating in the interventions. This study offers first-hand insights from parents into the perceived facilitators and barriers of implementing PACT-G at home in the immediate years after the completion of therapy. Identified facilitators highlight existing components of PACT-G that support parents’ use of the techniques; barriers identify areas that need further consideration. This study is one of the first to use implementation science frameworks with parents in the autism field and may provide a useful example for future studies.

## Supplemental Material

sj-docx-1-aut-10.1177_13623613221125630 – Supplemental material for Using implementation science frameworks to explore barriers and facilitators for parents’ use of therapeutic strategies following a parent-mediated autism intervention Supplemental material, sj-docx-1-aut-10.1177_13623613221125630 for Using implementation science frameworks to explore barriers and facilitators for parents’ use of therapeutic strategies following a parent-mediated autism intervention by Sophie Carruthers, Natasha Mleczko, Stephanie Page, Shalini Ahuja, Ceri Ellis, Patricia Howlin, Kathy Leadbitter, Lauren Taylor, Vicky Slonims and Tony Charman in Autism
