# Supplementary material for: Using implementation science frameworks to explore barriers and facilitators for parents’ use of therapeutic strategies following a parent-mediated autism intervention
Source: Autism. 2022 Oct 31;27(4):1011–25. doi: 10.1177/13623613221125630 (PMC10115939; doi:10.1177/13623613221125630)
Supplement: sj-docx-1-aut-10.1177_13623613221125630 – Supplemental material for Using implementation science frameworks to explore barriers and facilitators for parents’ use of therapeutic strategies following a parent-mediated autism intervention [file sj-docx-1-aut-10.1177_13623613221125630.docx]

**Supplementary Materials**

**Methods (additional details)**

The stopping point for data collection was informed by the following factors: the overlapping experiences of the sample (i.e., families with at least one child diagnosed with autism living in London), the framework-informed approach to data collection and analysis, the total possible sample of 41 families, the relatively broad study aim and the varied experiences of family life (Malterud, Siersma, & Guassora, 2016; Vasileiou, Barnett, Thorpe, & Young, 2018).

**Use of CFIR-ERIC mapping tool**

The CFIR-ERIC mapping tool organises the barriers according to CFIR construct. In order to use the tool, we therefore had to map each barrier identified by parents to the most relevant CFIR construct. Whilst some barriers already represented CFIR constructs, the barriers linked to TDF domains were mapped to the most closely related CFIR domain through a process of discussion among the researchers.

Then, the relevant CFIR domains were entered into the CFIR-ERIC Microsoft Excel tool. The tool produces a list of implementation strategies ranked most highly by the experts for the barriers specified. The developers of the tool emphasise how researchers should select and tailor strategies to address the specific context of implementation (Powell et al., 2017; Waltz et al., 2019). Therefore, to identify implementation strategies for further consideration, we considered the following: barriers that may represent priorities to target (e.g., most often reported or most likely to interfere with implementation) and the context of the parents’ implementation (i.e., at home with their child).

**Key term definitions:**

**Implementation Science**

“Implementation science is “the scientific study of methods to promote the systematic uptake of research findings and other EBPs into routine practice, and, hence, to improve the quality and effectiveness of health services.” Implementation science is distinct from, but shares characteristics with, both quality improvement and dissemination methods. Implementation studies can be either assess naturalistic variability or measure change in response to planned intervention. Implementation studies typically employ mixed quantitative-qualitative designs, identifying factors that impact uptake across multiple levels, including patient, provider, clinic, facility, organization, and often the broader community and policy environment. Accordingly, implementation science requires a solid grounding in theory and the involvement of trans-disciplinary research teams.” (Bauer, Damschroder, Hagedorn, Smith, & Kilbourne, 2015, p. 1)

**Implementation Strategy**

These are defined as methods or techniques that can be applied to support the adoption, implementation, and sustainability of evidence-based interventions, programmes or policies. The most recent and widely used taxonomy of implementation strategies within healthcare is the ERIC taxonomy – which was derived based on systematic evidence review and an expert consensus process. The ERIC taxonomy identifies 73 different implementation strategies that can be sued within healthcare settings (Powell et al., 2015).

**Table S1.**

A summary of the PACT-G therapy stages and therapeutic strategies that may be covered within each stage

| **PACT stage** | **Description** | **Therapeutic strategies that may be covered** |
| --- | --- | --- |

| 1. Establishing shared attention | The parent/LSA learns to sensitively observe the child’s focus, non-verbal and verbal signals and thereby to identify opportunities for shared attention. | - The child chooses - Following - Maintaining - Watching and waiting - Sharing the child’s interests - Mirroring - Positioning - Managing arousal levels |
| --- | --- | --- |
| 1. Synchronicity and sensitivity | This stage emphasises parent/LSA sensitivity in responding, with a focus on the child’s perspective and experiences. Parents/LSAs are encouraged to closely observe the child in order to identify opportunities to respond thereby reducing (i) asynchronous communication (mistimed responses which place a demand on the child) and (ii) increasing adult synchronous communication. | - Reduced demands on the child’s attention and processing - Reduced language demands - Reduced expectations - Matching the child’s pace - Commenting and re-phrasing - Acknowledging the child’s communication - Social routines and songs - Use of appropriate praise |
| 1. Focusing on language input | The parent/LSA selects and models language that accurately matches the child’s interests and communication competencies. Parent/LSA language and non-verbal gestures are carefully monitored and modified to be contingent with child’s focus and comprehension. The parent/LSA is trained to respond to the child’s non-verbal communication and to model complementary verbal responses that express the child’s inferred communication intent. | - Language Mapping and Language Modelling - using words/phrases that are: - Matched to child’s language level - Semantically Contingent - Salient - Consistent - Repeated - Supported by additional strategies - (Gesture, Pointing, Facial Expression) - Imitation (of child vocalisations/ word approximations) |
| 1. Establishing routines and anticipation | This is a consolidation phase that aims to develop child verbal understanding, anticipation and participation using repetitive rhymes, predictable routine phrases and familiar interactive play. | - Repetition - Anticipation - Pause - Imitation of words - Familiar routines/ rhymes - Repetitive scripts |
| 1. Increasing communication functions | Communication acts are elicited by the sensitive use of communication “teasers” to provide opportunities for child initiation. For example, the parent/LSA may make use of pauses and gaps within familiar, predictable play situations which the child fills with social and verbal responses. Communicative teasers entice the child to initiate intentional communication. These are gradually extended to pose deliberate problems and/or to introduce “sabotage” in situations where the parent/LSA makes obvious mistakes (e.g. offering an empty cup or unopened snack, or a puzzle/game with pieces missing). | - Breaks /pauses in familiar routines - Sabotage |
| 1. Language expansion/ extensions | Expands on the child’s understanding and use of language, thus elaborating on the child’s language repertoire, including extending vocabulary, phrases, sentences, the social use of language and developing to-fro conversation. | - Repeating back - Rephrasing - Recasts - Syntactic expansion - Semantic expansion - Elaboration - Conversation reciprocity - Conversational building and flow |

**Table S2. Topic guide**

| **Question no.** | **Question** | **Domain** |
| --- | --- | --- |
|  | *Do you currently use any of the strategies or techniques you learned during PACT-G with your child?* | ***Use of strategies*** |
| 2. | ***If the answer to 1 was yes:*** *Which of the PACT-G strategies do you use?*  ***If the answer to 1 was no:*** *It would be helpful to know why that is?* | ***Use of strategies*** |
| 3. | ***If the answer to 1 was yes:*** *How often do you use these strategies?*  ***If the answer to 1 was no:*** *When did you stop using them?* | ***Use of strategies*** |
| 4. | ***If the answer to 1 was yes:*** *Do you use the PACT-G strategies as much as you would like to?*  ***If the answer to 1 was no:*** *Before you stopped, were you using any of the strategies? Which strategies were you using?* | ***Use of strategies*** |
| 5. | ***If the answer to 1 was yes:*** *Why have you continued to use them?* | ***Use of strategies*** |
| 6. | In what situations/parts of your daily life do you use the PACT-G strategies? What about in the shops, in the park, during playtime, or mealtime? | **Environmental Context & Resources** |
| 7. | Are there any contexts or situations in which you find it easier to use the PACT-G strategies? Why are these situations easier? | **Environmental Context & Resources** |
| 8. | What about any situations in which it’s harder or more challenging to use them? What makes it challenging? (Prompts: time constraints, resource constraints, competing demands) | **Environmental Context & Resources** |
| 9. | What are the factors that influence the way in which or how often you use the strategies? What determines when you do or don’t use them? Prompts: preferences or needs of your child, other children or adults in the home, your daily and weekly routine, family circumstances, life events? | **Environmental Context & Resources** |
| 10. | This first one sometimes feels like a bit of a quiz, but we’re just interested in whatever it is you think. There’s no right or wrong answer. PACT-G therapy helps parents change how they interact with their child, such that they follow their child’s lead more often – and this idea was central to the therapy. Why do you think the therapy focuses on this? | **Knowledge** |
| 11. | Do you use any other guidance or training to support your child’s communication? | **Knowledge** |
| 12. | How does PACT-G compare to other therapies or training you’ve previously been offered or completed? | **Knowledge** |
| 13. | If we think about the format of the therapy (e.g. for the therapist to visit you at your home and over skype, and provide feedback on videos etc.) How did you find that? | **Environmental Context & Resources** |
| 14. | Did you have any of the home-school meetings during the trial? Do you think speaking with the TA during those meetings influenced your experience as a parent of PACT-G at all? | **Social role and identity** |
| 16. | Are the PACT-G strategies easy to learn? | **Memory, attention, decision making** |
| 17. | What skills do you think you need to use the strategies that (*insert therapist’s name*) was helping and encouraging you to use with your child? | **Skills** |
| 18. | Do you think that the PACT-G therapy helped you to learn these skills? | **Skills** |
| 19. | Are these skills still important to you as a parent? | **Skills** |
| 20. | To support communication in young children, the PACT-G therapists work directly with you as parents, rather than directly with the children. How do you feel about this approach? For instance, as opposed to working directly with the children or only working with the teaching assistants? | **Social role and identity** |
| 21. | What do you think is the value of using the strategies with your child? For instance, what sort of outcomes do you expect? Prompt: One way of thinking about this is what do you think will happen if you don’t use the strategies with your child? | **Optimism** |
| 22. | What did or do you want to achieve by using the PACT-G strategies? | **Goals** |
| 23. | How do you feel about PACT-G and the strategies? | **Emotions** |
| 24. | Did your emotions towards PACT-G change at any point over the course of the trial and/or since the trial? | **Emotions** |
| 25. | How confident are you that you can use the PACT-G strategies during interaction with your child? | **Beliefs about capabilities** |
| 26. | Do you find yourself using the strategies automatically, or do you need to be reminded or consciously think about it? | **Behavioural regulation** |
| 27. | Did you ever or do you tend to make any plans in your mind around when you’re going to use any of the PACT-G strategies or how you’re going to use them? | **Intentions** |
| 28. | If you or another parent wanted to increase the amount of time spent using the strategies, what do you think you or they should do? | **Behavioural regulation** |
| 29. | As we know, life can be very busy. Among all of the other things you need to do in your daily life, where does using PACT-G strategies come in the priorities of things to do? | **Goals** |
| 30. | When people do things, sometimes they can be made to feel good about it and they want to do it again and sometimes they can feel a bit negative about it and are discouraged from doing it again. When you use the strategies from PACT-G, is there anything that can make you want to use them again? (prompt i.e. anything that makes you feel good for having tried to use the strategies?) | **Reinforcement** |
| 31. | What about anything that makes you not want to use them again? | **Reinforcement** |
| 32. | How do family and friends feel about what you are trying to achieve with PACT-G? | **Social influences** |
| 33. | Have you tried to encourage anyone else to use the PACT-G strategies with [insert child’s name]? Can you tell me a bit about that? | **Social influences** |
| 34. | Have you ever spoken with other parents of autistic children about therapies or training you or they have received, PACT-G or otherwise? Could you tell me a bit about that. Do you think hearing about other training or therapies encourages you or discourages you from using PACT-G strategies? | **Social influences** |
| 35. | What impact do you think using the strategies has had on your child? | **Beliefs about consequences** |
| 36. | Have these effects spread to other people or settings? | **Beliefs about consequences** |
| 37. | What sort of impact do you think PACT-G has had on you? | **Beliefs about consequences** |
| 38. | Has it had an impact on your relationship with your child? | **Beliefs about consequences** |
| 39. | And finally, do you think PACT-G as a therapy met your needs? | **Beliefs about consequences** |

**Table S3. Coding framework (TDF)**

| **TDF (Version 2)** | | | |
| --- | --- | --- | --- |
| **Domain** | **Definition** | **Component constructs** | **Examples for this study** |
| **Knowledge** | An awareness of the existence of something | Knowledge (including knowledge  of condition/scientific rationale)  Procedural knowledge  Knowledge of task environment | *Knowledge of PACT-G, the philosophy/theory behind it, the strategies it uses, autism generally, other therapies or key related information* |
| **Skills** | An ability or proficiency acquired through practice | Skills  Skills development  Competence  Ability  Interpersonal skills  Practice  Skill assessment | *Skills that are needed for PACT-G* |
| **Social/professional role and identity** | A coherent set of behaviours  and displayed personal qualities of an individual in a social or work setting | Professional identity  Professional role  Social identity  Identity  Professional boundaries  Professional confidence  Group identity  Leadership  Organisational commitment | *The adult’s ‘role’ as a parent and how this interacts with PACT-G, their child, their child’s autism or the use of strategies* |
| **Beliefs about capabilities** | Acceptance of the truth, reality or validity about an ability, talent or facility that a person can put to constructive use | Self-confidence  Perceived competence  Self-efficacy  Perceived behavioural control  Beliefs  Self-esteem  Empowerment  Professional confidence | *Self-confidence, perceived competence, self-efficacy, self-esteem, beliefs, empowerment, perceived behavioural control, e.g. not feeling confident, worrying they’re not doing it right* |
| **Optimism** | The confidence that things will  happen for the best or that  desired goals will be attained) | Optimism  Pessimism  Unrealistic optimism  Identity | *The value of using PACT-G strategies, what might they lead to, what they hope will come as a result* |
| **Beliefs about consequences** | Acceptance of the truth, reality,  or validity about outcomes of a  behaviour in a given situation | Beliefs  Outcome expectancies  Characteristics of outcome  expectancies  Anticipated regret  Consequents | *Perceived impact, beliefs about impact, regret, knock-on effects associated with PACT-G, changes of attitudes or mindsets, understanding their child or their autism better* |
| **Reinforcement** | Increasing the probability of a  response by arranging a  dependent relationship, or  contingency, between the  response and a given stimulus | Rewards (proximal/distal, valued/not  valued, probable/improbable)  Incentives  Punishment  Consequents  Reinforcement  Contingencies  Sanctions | *Anything that feeds into whether they feel good or bad about using the PACT-G strategies or their participation in the trial* |
| **Intentions** | A conscious decision to perform a behaviour or a resolve to act in a certain way | Stability of intentions  Stages of change model  Transtheoretical model and  stages of change | *Plans made around strategy use* |
| **Goals** | Mental representations of  outcomes or end states that an  individual wants to achieve) | Goals (distal/proximal)  Goal priority  Goal/target setting  Goals (autonomous/controlled)  Action planning  Implementation intention | *Targets, what they want to achieve, what were they aiming for by participating in PACT-G, what they want their child or family to gain* |
| **Memory, attention and decision processes** | The ability to retain information, focus selectively on aspects of the environment and choose between two or more alternatives | Memory  Attention  Attention control  Decision making  Cognitive overload/tiredness | *How easy the strategies were to learn or remember* |
| **Environmental context and resources** | Any circumstance of a person’s  situation or environment that  discourages or encourages the  development of skills and  abilities, independence, social  competence and adaptive  behaviour | Environmental stressors  Resources/material resources  Organisational culture/climate  Salient events/critical incidents  Person Å~ environment interaction  Barriers and facilitators | *Using the strategies in everyday life. How the aspects of their context affects the use of strategies (that are not coded elsewhere). Things that make it harder or easier. People, places, time of day* |
| **Social influences** | Those interpersonal processes  that can cause individuals to  change their thoughts, feelings,  or behaviours | Social pressure  Social norms  Group conformity  Social comparisons  Group norms  Social support  Power  Intergroup conflict  Alienation  Group identity  Modelling | *How others feel about them using the strategies, about PACT-G, about therapy for their child or about their child’s autism* |
| **Emotion** | A complex reaction pattern,  involving experiential,  behavioural, and physiological  elements, by which the  individual attempts to deal with  a personally significant matter  or event | Fear  Anxiety  Affect  Stress  Depression  Positive/negative affect  Burn-out | *Emotions and feelings associated with PACT-G, the therapist, the strategies or their child’s autism* |
| **Behavioural regulation** | Anything aimed at managing or  changing objectively observed or measured actions | Self-monitoring  Breaking habit  Action planning | *Is use of the strategies automatic, or needs to reminding/conscious thought to use them? What would they need to do to use them more often?* |

**Table S4.** Coding framework (CFIR)

| **CFIR** | | | |
| --- | --- | --- | --- |
| **Domain** | **Code** | **Definition** | **Subcodes** |
| **CFIR Intervention Characteristics** | **Intervention Source** | Parents’ perceptions of the legitimacy of the team who developed PACT-G |  |
|  | **Evidence Strength & Quality** | Parents' perceptions of the quality and validity of evidence supporting the belief that the PACT-G therapy will have desired outcomes |  |
|  | **Relative Advantage** | Parents’ perceptions of the advantage of implementing the PACT therapy versus an alternative solution |  |
|  | **Adaptability** | The degree to which PACT-G can be adapted, tailored, refined, or reinvented to meet particular child, family and school needs. |  |
|  | **Trialability** | The ability to try out PACT-G strategies on a small scale and to be able to stop or modify as needed. |  |
|  | **Complexity** | Perceived difficulty of the PACT-G therapy, reflected by duration, scope, disruptiveness, and intricacy and number of steps required to implement. |  |
|  | **Design Quality & Packaging** | Parents’ views on how well the PACT-G therapy is bundled, presented, and assembled (including format of the therapy). |  |
|  | **Cost** | Costs associated with implementing the PACT-G therapy i.e. costs to the parent (time, energy, money). |  |
| **CFIR Outer Setting** | **School** | The nature of the relationship with the child's school and ways in which the school influenced implementation of PACT-G at home |  |
|  | **Child characteristics** | The needs and characteristics of the child and how these influence the parents’ implementation of PACT-G | Child age*  Child behaviour*  Child mood*  Child interests*  Child needs* |
| **Use of strategies** |  | This code captures the parent’s responses to the first set of questions in the interview about the extent to which they still used the strategies. Information is captures on which strategies are mentioned, how often they are used, how they’re used and whether they’re used as often as they would like. If parents were no longer using the strategies, reasons for stopping were also captured under this code. | Which strategies used?*  How often?*  How strategies were used?*  Used as often as wanted?*  Reasons for stopping* |

*Note.*

*Emerged from deductive coding.

**Table S5.** A summary of the barriers and facilitators within the theme of Motivating Factors divided into the two subthemes of Compatibility and Buy-In and Alignment of Goals and Outcomes with quotes.

| **Subtheme 1: Compatibility and Buy-In** | |
| --- | --- |
| **Facilitators** | |
| **Belief** | **Quote** |
| I’m confident using the strategies | *“Interviewer: How confident are you that you can use the PACT-G strategies during interaction with your child?*  *Parent: I'm ten out of eight.”* |
| I feel very positive about the PACT-G strategies | *“Those techniques, it’s like you have a little box of magic and suddenly everything changes, so everyone should know…So, I’m still happy and so grateful for everything I’ve learned.”* |
| The PACT-G strategies have advantages over other therapies | *“It’s quite useful because you don’t get a lot of input…most of the therapy has gone into school…it’s kind of nice to have some hands-on guidance. That’s quite rare with parents, you don’t really get a lot of direct training.”* |
| Training parents to use the strategies with their children is the right approach | *“It was directly with me and I am implementing what I learn which is the right way because if I don’t…if she doesn’t work with me because she’s with us a few hours then she leaves, we are the ones are [going to] follow what we’ve learned every second. So yeah, that was the right way. She is teaching me how to be with my child.”* |
| I believe in the principles of PACT-G | *“I strongly believe in [PACT-G] because follow the lead is so important, it’s crucial in life because you give them…confidence…you are there…to follow their lead, their needs…They become more confident.”* |
| Video feedback provided me with a secure understanding and knowledge of the strategies | *“Yeah, that was really great to be honest. Sometimes I didn’t realise how I behaved and then we watched the film and I realised I did something that wasn’t correct, it wasn’t based on the education she teach me…how to behave with my child. That was like a window to our communication.”* |
| The therapists were wonderful teachers and provided a lot of support | *“I had a lovely therapist who I enjoyed to spend time with and my son as well”* |
| **Barriers** | |
| You need to persevere because progress can be slow | *“They’re not going to work overnight. So, you just have to keep doing it and hopefully it’s going to work.”* |
| I wasn’t so positive about the therapy at the start | *“I struggled [to take on the role of the therapist] in the beginning. Where I come from a child has…one-to-one sessions with a speech and language therapist. So, in the beginning I was not happy with it. But…at the end I think it is the right way to do it because…the first therapist living with the child is the mum or the parent…If the therapist is doing one hour a week with good communication and then the rest of the week nothing is happening…it does not create consistency, it just confuses the child.”* |
| Some strategies go against my natural parenting instincts | *“I always try and learn not to correct him too quickly, because I remember we focused on that a lot during the therapy…let him take the lead, try…not to correct. It’s just a general instinct, because obviously if he does something incorrectly…your instinct is to correct and teach.”* |
| I lack confidence using the strategies | *“Interviewer: How confident are you in using the strategies?*  *Parent: Not very confident but at least I know them, and I know the benefits of them. I wouldn’t say that I’m 100% confident now but better than before.”* |
| PACT-G is too easy for my child | *“He’s much smarter than what they are giving him…like the session they give him, like toys, they put them on the table.”* |
| Other therapies are more suitable for my child | *“I started to use with him these ABA techniques and what I feel is the best for him.”* |
| **Subtheme 2: Alignment of Goals and Outcomes** | |
| **Facilitators** | |
| I want my child to communicate better | *“I think what I wanted to achieve was I wanted him to be able to communicate his needs and his interests.”* |
| I want my child to be more independent and have a better quality of life | *“I just want to achieve to understand my child more, for him to get more out of life, to just get it easier to work between us, to get him more out of his shell more, to get more interaction.”* |
| Using the PACT-G strategies is still a top priority for me | *“It’s…big priority because our daughter obviously most important thing for us as a parent, so dealing with her is very important and if she have any improvements, we are happy, we are over the moon... so PACT-G is important by more than a lot of things in our lives actually, the most important thing.”* |
| I wanted to learn how to support my child (independently from professionals) | *“…being trained to support my child myself at home instead of relying on professionals all the time”* |
| My relationship with my child has improved | *“[The strategies] really just gives the parent a different sort of thing to try and use to make contact, to bond, and to teach... To realise things about their own child that they might have missed…my child will often talk with his eyes but it can be so quick sometimes you don’t realise that he’s looked over there and already picked something he wants to play with.”* |
| My child is more confident and engaged with things around him/her | *“He is making choices, he is saying what he wants and he is engaging more than before…he knows what he wants more and if he wants to play he just initiates and he comes to me and cuddles his dad and he’s interacting more with us”* |
| My wellbeing has improved | *“I think one of the blockers for me was that I was just too anxious about trying to get my child to talk and I think what he needed was time to just…do it on his own terms. I think PACT-G helped me win over that anxiety. There’s [now] a lot less anxiety on my part and I think my child can feel it as well because [our relationship] becomes a lot more relaxed.”* |
| I learn things about my child through the strategies | *“It was like a waking up call for me to be honest…I just found out things that my child was able to do or could not do really and…everyday was like full of surprises because I never knew, you know, like she’s able or knew some words or…by looking at things, like pictures and stuff like that.”* |
| My child’s wellbeing has improved | *“To be more in tune with him is also beneficial because it helps him being less stressed, so he felt a bit more understood…I think there’s less frustration on his part…when he’s home, he has…less tantrums, he’s not quite as aggressive”* |
| My child’s communication has improved | *“It was literally stepping back and sitting on my hands almost to stop myself...interfering almost in, in his play, and sort of being aware and being observant, and exploring how he might then take the lead. That encouraged then his sort of communication in leaps and bounds really.”* |
| I have learned to let go and accept things about my child’s autism | *“I remember he had a…pretend cake. When we started [PACT-G] I was desperately trying to get my child to slice this cake…trying to make him be the child that I wanted him to be. All he wanted to do was tap the underside of the cake...so the therapist said…‘tap it with him’. When I was trying to get him to slice the cake, he was just…pushing me away...but when I tapped the underside of the cake with him…he was totally engaged with me and he was looking at me, laughing, and he enjoyed it...I realised...he doesn't have to do things the way I think he should do them...it doesn't mean that it's not playing properly.”* |
| I’m now aware of how adjustments in my behaviour can help my child | *“Before the therapy I thought my child didn’t like me because she didn’t want to interact…but the therapist said…‘she wants you to participate, but in her time, not in your time’. Now…I sit on the floor and she just plays along…Sometimes, before [PACT-G]…I would try to teach her to play…and she [would become] upset….Now I know…she just wants me to sit down, or sometimes if she wants to really play, she’ll [put] something in my hand…so I understand now, it’s her play, not my play. Let her lead.”* |
| The wider family benefits from me using the strategies | *“She never used to interact [but now] she’s comes and she hugs me, tells me what she wants to eat, especially with her grandma and grandpa nowadays saying I want to sleep with you grandma or I want to go outside to the park with you, or I want ice cream, now she’s interacting with them so they are also happy.”* |
| Seeing the progress in my child makes me want to continue using the strategies | *“When she responds to what you’re saying. For example, if I’m having a play time with her and I see she’s coming closer and she enjoys it. So that…I feel good, makes me use it more…If I see a smile on her face it shows me that…what I’m doing is quite, you know, it’s useful and good, so I carry on using it.”* |
| **Barriers** | |
| PACT-G is less of a priority for me now | *“But now I can say she’s very comfortable with it, she comes herself and she tells [me] everything, she comes [to me] herself …and she tells me everything so the priority has become a bit less because she is already doing the work I want her to do.”* |
| My child has other needs that demand my energy and attention | *“Sometimes other things [the intention to use the strategies] just slip away because sometimes he doesn’t sleep for two or three nights and then he’s aggressive, angry and you can’t do [the strategies].”* |

**Table S6.** A summary of the barriers and facilitators within the theme Opportunity and Support with quotes.

| **Belief** | **Quote** | |
| --- | --- | --- |
| **Facilitators** | | |
| The strategies were easy to learn | | *“Interviewer: Are the PACT-G strategies easy to learn?*  *Parent: Yes, very easy.”* |
| I enjoyed working with the school | | *“I’ve been liaising with her teachers, and I try and keep the strategies they’ve been using at school at home so we’re both on the same page. So it sort of works both ways, to keep her calm at school and keep her calm at home.”* |
| I can incorporate my child’s interests when using the strategies | | *“So this is the thing that I learned…it’s going to be more interest for him, if I say “…just do this, do that", it’s just going to be…more like command and it’s better if he is doing by his own interest so it’s going to be more helpful for him, and as I sitting down with him and showing him I am here to support you if you want anything, just tell me.”* |
| My family and friends are supportive of using PACT-G strategies | | *“My brother helps me do the strategy…he just gives me the emotional support, so I just use that.”* |
| I can easily adapt the strategies to suit our needs or the situation | | *“It was something that you can take away and apply to most situations, and it was simplistic enough to incorporate into your daily lives.”* |
| **Barriers** | | |
| My child’s behaviour and mood influences when I can use the strategies | | *“[Using the strategies] is more challenging when he’s…very stuck…he becomes rigid…At some point, he’s more difficult to interact with him or to use the strategies in some circumstance…and sometimes he gets frustrated to use the strategy.”* |
| I need to accommodate my child’s specific needs | | *“It just has to be in quiet times when he’s not kind of feeling pressured.”* |
| I need time to do the strategies, which I don’t always have spare | | *“It’s just you have to be determined to learn it as well because sometimes you can be quite busy with other children and so [you need to] take that time out to do it. Sometimes it isn’t easy.”* |
| My family/friends do not get involved in using the strategies | | *“To be honest with you we don’t have a lot of friends, we have family that comes over and say ‘oh she improved’? But they’re not involved in PACT-G, maybe they don’t know we do it to be honest.”* |
| I didn’t have a strong relationship with the school at the time of the trial | | *“We didn’t work on those things together…we didn’t work with the school so they did it themselves and we did ourselves at home…I have no contact with anyone”* |

**Table S7.** A summary of the barriers and facilitators within the theme Parental Characteristics with quotes.

| **Belief** | **Quote** | |
| --- | --- | --- |
| **Facilitators** | | |
| I understand the philosophy of PACT-G | | *“To really…get to know your child…be in tune with your child’s needs…trying to understand your child better…find ways to communicate…even if it’s not verbal, like looking for those ‘little finds’ that they give you, the non-verbal cues. Because I think at the beginning…it was more about us…trying to get him to speak…and then [PACT-G] taught me actually that there’s more to it than just verbal communication.”* |
| Being patient is important | | *“It’s all about being patient with your child”* |
| The strategies are now automatic and spontaneous | | *“Obviously at first, I would need to think…how to play with him…but now it’s automatic, I don’t even…think about any strategies I know, I just interact and play with him automatically.”* |
| I’m open to thinking about how to change my behaviour | | *“Interviewer: Do you think there are any skills that you need to have to be able to learn and use these strategies?*  *Parent: […]allowing yourself to make mistakes.”* |
| Self-motivation is important | | *“I don’t think you need particular skills except just that you want to do it.”* |
| I plan when I’m going to use the strategies | | *“I will plan, maybe tomorrow I will sit down with him for 10 minutes, try to engage him with toys and…writing on paper, yeah I plan.”* |
| **Barriers** | | |
| Sometimes I forget to use the strategies | | *“It’s really easy but when you want to do it and you have an autistic child, it’s a challenge. You constantly forget and you need someone to remind you to do that.”* |
| The strategies sometimes require conscious effort and thought | | *“I really have to…remind myself about his state, you know, sometimes we are in a hurry and [I] try to [show] him the urgency while he’s doing his own things and I get really frustrated but then I try to keep in my mind that he needs time to process, to get used to a different routine”* |

**Table S8.** Barriers mapped to CFIR constructs and the ten highest ranked implementation strategies recommended by the CFIR-ERIC mapping tool.

| **Barrier** | **Original TDF/CFIR domain** | **Mapped CFIR construct for the CFIR-ERIC tool** | **Top ten suggested strategies identified by CFIR-ERIC mapping tool**  **(Cumulative endorsement %)** |
| --- | --- | --- | --- |
| I wasn’t so positive about the therapy at the start (but my feelings about PACT changed) | Emotion | Characteristics of Individuals (Individual Stage of Change) | 1. Identify and prepare champions (Cumulative percentage: 249%) 2. Assess for readiness and identify barriers and facilitators (220%) 3. Conduct local needs assessment (218%) 4. Build a coalition (200%) 5. Conduct local consensus discussion (191%) 6. Capture and share local knowledge (182%) 7. Create a learning collaborative (166%) 8. Conduct educational meetings (164%) 9. Alter incentive/allowance structures (157%) 10. Promote network weaving (146%) |
| Progress can be slow (so remaining positive and persevering is important) | Optimism | Characteristics of Individuals (Knowledge & Beliefs about the Intervention) |  |
| The PACT-G strategies go against my parenting instincts | Social Role and Identity | Characteristics of Individuals (Self-efficacy) |  |
| I lack confidence using the strategies | Beliefs about Capabilities | Characteristics of Individuals (Self-efficacy) |  |
| PACT-G is too easy for my child | CFIR Outer Setting (Child) | Outer Setting (Patient Needs & Resources) |  |
| There are other therapies that are more suited for my child | CFIR Intervention Characteristics – Relative Advantage | Intervention Characteristics (Relative Advantage) |  |
| PACT-G isn’t a top priority for me | Goals | Inner Setting (Relative Priority) |  |
| My child has other needs that demand my energy and attention | CFIR Outer Setting (Child) | Outer Setting (Patient Needs & Resources) |  |
| My child’s behaviour influences when I can use the strategies | CFIR Outer Setting (Child) | Outer Setting (Patient Needs & Resources) |  |
| It depends on what mood my child is in | CFIR Outer Setting (Child) | Outer Setting (Patient Needs & Resources) |  |
| I can only use the strategies if I accommodate my child’s specific needs and preferences | CFIR Outer Setting (Child) | Outer Setting (Patient Needs & Resources) |  |
| I have other demands on my time and sometimes I don’t have any left for the strategies | Environmental Context & Resources | Inner Setting (Available Resources) |  |
| My family/friends do not get involved in using the strategies | Social Influences | Inner Setting (Networks & Communications) |  |
| I didn’t have a strong relationship with the school at the time of the trial | CFIR Outer Setting (School) | Outer Setting (Cosmopolitanism) |  |
| You need time and to not be in a rush | Environmental Context & Resources | Inner Setting (Available Resources) |  |
| Sometimes I forget to use the strategies | Memory, Attention & Decision Processes | Process (Planning) |  |
| The strategies sometimes require conscious effort and thought | Behavioural Regulation | Characteristics of Individuals (Individual Stage of Change) |  |

*Note.*

Each barrier was mapped to a CFIR construct for the purpose of this exercise. Definitions for each CFIR construct in relation to barrier are provided in Table A.6. This percentages are based on endorsements from n=169 implementation science experts who selected and ranked "up to 7 strategies that would best address" each CFIR barrier (Waltz et al., 2019). The cumulative percentages represent the sum of the individual endorsements for each strategy across all barriers (i.e. CFIR constructs) selected. Percentages for each individual CFIR construct are provided in Table A.6.

CFIR = Consolidated Framework for Implementation Research, ERIC = Expert Recommendations for Implementing Change, PACT-G = Paediatric Autism Communication Trial – Generalised, TDF = Theoretical Domains Framework

**Table S9.** Implementation strategies identified as target areas for further exploration.

| **Implementation Strategy** | **Definition** | **Example barriers that may be addressed** |
| --- | --- | --- |
| Assess for readiness and identify barriers and facilitators | Assess various aspects of an organization to determine its degree of readiness to implement, barriers that may impede implementation, and strengths that can be used in the implementation effort | - I have other demands on my time and sometimes I don’t have any left for the strategies - The PACT-G strategies go against my parenting instincts - PACT-G isn’t a top priority for me |
| Conduct local needs assessment | Collect and analyze data related to the need for the innovation | - PACT-G is too easy for my child - There are other therapies that are more suited for my child |
| Promote network weaving | Identify and build on existing high-quality working relationships and networks within and outside the organization, organizational units, teams, etc. to promote information sharing, collaborative problem-solving, and a shared vision/goal related to implementing the innovation | - I can only use the strategies if I accommodate my child’s specific needs and preferences - It depends on what mood my child is in - My child’s behaviour influences when I can use the strategies |
| Conduct educational meetings | Hold meetings targeted toward different stakeholder groups (e.g., providers, administrators, other organizational stakeholders, and community, patient/consumer, and family stakeholders) to teach them about the clinical innovation | - My family/friends do not get involved in using the strategies |

*Note.*

The example barriers are illustrative of which of the 17 barriers reported by parents may be addressed by each implementation strategy. The list is not intended to be exhaustive and other barriers may also be supported by these strategies. PACT-G = Paediatric Autism Communication Trial - Generalised

**References**

Bauer, M. S., Damschroder, L., Hagedorn, H., Smith, J., & Kilbourne, A. M. (2015). An Introduction to Implementation Science for the Non-Specialist. *BMC Psychology, 3*, 32. doi:10.1186/s40359-015-0089-9

Malterud, K., Siersma, V. D., & Guassora, A. D. (2016). Sample Size in Qualitative Interview Studies: Guided by Information Power. *Qualitative Health Research, 26*(13), 1753-1760. doi:10.1177/1049732315617444

Powell, B. J., Beidas, R. S., Lewis, C. C., Aarons, G. A., McMillen, J. C., Proctor, E. K., & Mandell, D. S. (2017). Methods to Improve the Selection and Tailoring of Implementation Strategies. *Journal of Behavioral Health Services and Research, 44*(2), 177-194. doi:10.1007/s11414-015-9475-6

Powell, B. J., Waltz, T. J., Chinman, M. J., Damschroder, L. J., Smith, J. L., Matthieu, M. M., . . . Kirchner, J. E. (2015). A Refined Compilation of Implementation Strategies: Results from the Expert Recommendations for Implementing Change (Eric) Project. *Implementation Science, 10*(1), 21. doi:10.1186/s13012-015-0209-1

Vasileiou, K., Barnett, J., Thorpe, S., & Young, T. (2018). Characterising and Justifying Sample Size Sufficiency in Interview-Based Studies: Systematic Analysis of Qualitative Health Research over a 15-Year Period. *BMC Medical Research Methodology, 18*(1), 148. doi:10.1186/s12874-018-0594-7

Waltz, T. J., Powell, B. J., Fernandez, M. E., Abadie, B., & Damschroder, L. J. (2019). Choosing Implementation Strategies to Address Contextual Barriers: Diversity in Recommendations and Future Directions. *Implementation Science, 14*(1), 42. doi:10.1186/s13012-019-0892-4
